# Interaction between image and text during the process of biblical art reception

**DOI:** 10.16910/jemr.13.2.14

**Published:** 2021-03-12

**Authors:** Gregor Hardiess, Caecilie Weissert

**Affiliations:** University of Tübingen, Tübingen, Germany

**Keywords:** Eye movement, eye tracking, contextual relevance of text, top-down processing, attentional control, art perception, region of interest

## Abstract

In our exploratory study, we ask how naive observers, without a distinct religious background, approach biblical art that combines image and text. For this purpose, we choose the book ‘New biblical figures of the Old and New Testament’ published in 1569 as source of the stimuli. This book belongs to the genre of illustrated Bibles, which were very popular during the Reformation. Since there is no empirical knowledge regarding the interaction between image and text during the process of such biblical art reception, we selected four relevant images from the book and measured the eye movements of participants in order to characterize and quantify their scanning behavior related to such stimuli in terms of i) looking at text (text usage), ii) text vs. image interaction measures (semantic or contextual relevance of text), and iii) narration. We show that texts capture attention early in the process of inspection and that text and image interact. Moreover, semantics of texts are used to guide eye movements later through the image, supporting the formation of the narrative.

## Introduction

The notion that eye movements mostly behave under cognitive, i.e. task-driven and top-down control, as well as the investigation of such information acquisition behavior in conjunction with the perception of art dates back to the pioneer work of Yarbus in the ’70s (1). Top-down mechanisms imply that the visual input is analyzed in a way that is driven by the observer’s experience, pre-knowledge, and goals (task-driven attention). With his research, Yarbus demonstrated that the process of active vision (2) could certainly be guided by internal goal(s) and that these can be influenced and/or generated by external task instructions (3, 4, 5). Contrary to top-down, bottom-up control implies that features and the characteristics of the visual stimulus itself, i.e. the saliency of the stimulus, will guide attention and thus eye movements (stimulus-driven attention). Nowadays, the interaction between bottom-up and top-down processes (a comprehensive overview related to art can be found at Pelowski et al. (6)) that operate at different levels of the viewer’s experience in order to control eye movements is well investigated in the literature (e.g. 7, 8). By now it has also been well established that beside the internal task, emotional states and the person’s culture and expertise also affect the individual scanning behavior by shaping the behavioral goal(s) (e.g. 9, 10, 11, 12).

Besides externally provided verbal instructions, as in Yarbus’ experiments (1), text within the visual stimulus (e.g. in comic-like artwork) or accompanying it (e.g. text descriptions of artwork in museums or booklets) can carry contextual or semantic information guiding the receptive, gaze shifting behavior as well. Inherently, text in scenes (e.g. depictions of signs, banners, advertisement billboards, license plates, and others) disproportionately attracts attention when real world, artificial or pictorial sceneries are inspected (13, 14). The origin of such an attentional bias is still debated and explanations range from pure bottom-up processing of artwork features (e.g. color, form, attractiveness (15, 16)) to theories of top-down, i.e. contributions of memory, personality, context, and cognition (e.g. 14, 17, 18, 19). A plausible intermediate approach seems to be that the attentional capture originating from texts could be driven by some particular classes of features (or objects) which attract gaze independently of their low-level visual characteristics. Here, text may provide speciﬁc features, similar to faces that attract attention but differ from the features that are typically associated with visual saliency (13).

A potent method to investigate the interaction of vision and semantically meaningful information carried by text or language is the tracking of gaze movements (i.e. gaze = head + eye) while human observers are looking at visual stimuli and simultaneously reading texts or listening to speech. This allows for a real-time analysis of the behavioral consequences of such integrated information sources due to the semantic guidance of visual attention (e.g. 20, 21). Once such narrations have been captured and formed, they heavily influence saccadic decisions for the further inspection of the visual stimuli due to top-down factors (22). As mentioned above, texts or captions itself attract attention (i.e. observers spend a large proportion of time reading), regardless of their content of information or degree of redundancy (13, 14, 22). Such behavior seems to be inappropriate, since these unnecessary gaze shifts waste time and lead to the image (or video) being unattended to, with the risk of losing information. The interpretation of this is that people have a habit of reading text because our prior experiences have taught us that text often conveys important information. In addition, observers are continually judging the value of information coming from all available sources (23). Therefore, when text is provided, it is considered potentially important (i.e. informationally-dense and reliable) and guides the line of sight into its region. In our study, we ask whether this also pertains to historical media that combine image and text.

### The Present Study

During early modern times artistic genres reached a great blossoming, which intentionally unite image and text (24). With the printed vernacular editions of the Bible, e.g. also a pictorial translation encompassing the entire Old and New Testament started throughout Europe in the early sixteenth century (25). Word and image coexist here, and the reader was and is expected to read the text as well as to look at the images and to be able to connect and to relate word an image to each other mentally or verbally (24, 26). Depending on previous knowledge, level of education and interests, this will have been possible with varying degrees of intensity. Recipients who were interested in these works of art - most of them prints - were accustomed to the combination of word and image and regarded the juxtaposition of image and text as a gain. In this respect it is worth to note that the fifteenth- and the sixteenth-century Netherlands and Germany were among the most literate societies in the world and the ability to read was very high (27, 28, 29).

The most important and most read book of the 16th century was the Bible, the Old and New Testament. Especially popular were Bibles with illustrations, belonging to the genre of illustrated Bibles (30). During this time, the Bible became the center of faith and the most important book for an individual approach to religious belief for the laity (31). One of the popular illustrated Bible was published in 1569 in Frankfurt am Main containing woodcuts by Jost Amman (1539-1592) based on drawings by Johann Melchior Bocksberger (1530-1589). This Bible was lavishly illustrated with woodcuts showing large, expressive figures in the foreground and a detailed and lively environment of further scenes, figures and animals. The woodcuts are not limited to a literal translation of Bible paraphrases but provide a great surplus of visual stimuli and narrative and copious additional details and ornaments. They depict scenes from the Old and New Testaments and are combined with texts (in Latin and old German). These texts are short paraphrases of the Bible’s narrative in rhymed form. Depending on personal interest and education, the texts could be read or neglected, since the images are not necessarily dependent on the texts. Because of the loose relationship between image and text, this book is also suitable for a study of present-day readers, with a European educational background who can decide for or against reading the text during their contemplation of the woodcuts.

Since contemporary sources from the 16th century that provide concrete information on the reading behavior of the lay public are very scarce, art historians are on the search for methods that allow a better understanding in respect to the semantic and contextual distribution during the viewing process. We certainly cannot take the historical viewer's perspective and we cannot reconstruct it with all its physical and mental implications. Nevertheless, we can raise the matter how text and image were and are mentally processed. Studies could show that fundamental mental processes have remained the same over the time and that they are based on biological and neural processes (32, 33).

In the process of engagement with art, content was shown as a relevant dimension (34, 35, 36, 37). On the one hand, content can be understood as a reference to objects and situations depicted in the artwork (34, 38). However, content may also refer to the artwork’s meaning which is expressed in the depicted situation (for a more comprehensive consideration of content in the framework of pictorial art, see Commare et al. (35)). The semantic content (i.e. meaning) is a crucial factor in guidance visual attention for observers looking at pictorial scenes (including artwork). Here, the pictorial stimulus itself contains much of such information which originates from prior knowledge and memory formed through experience and learning (39, 40), e.g. inferring that the location of an unknown driving object to likely be near the ground, or expecting a toothbrush to be in the bathroom rather than in the kitchen. In a recent review, Wu et al. (41) sub-divided such semantic and contextual content into four domains: i) information providing the gist of a scene, ii) information about scene-object relations and iii) object-object relations, and iv) conceptual-semantic associations between objects in the scene. A similar framework regarding the process of viewing artwork is provided in Harland et al. (42). Here, the gist (established within the first 100 ms) provides a rough articulation of pictorial, structural, and semantic properties of the scene and is followed by exploratory eye movements in order to establish the spatial and semantic relationships between objects (42). The ability to encode the semantic content of an artwork on a higher processing stage (43) is clearly dependent on the declarative knowledge of the recipient. This kind of knowledge permits the classification of iconographic contents (cf. (44)) during a later and deliberate processing phase (45) and enables an interpretative and interactive engagement with the artwork. Here, the depth of a person’s declarative knowledge correlates with the ease of recognizing and interpreting the semantic content and thus the meaning (35, 45, 46).

Therefore, we assume that research that deals with the production and reception of historical art could learn from empirical studies more about the meaning of the connection of word and image and receive fruitful advice for methodological approaches to the analysis of these image-text genera. The basis of the present study is to consider these accounts based on empirical investigations in terms of eye movement behavior, since eye tracking studies were already used successfully to investigate the visual behavior of observers (from novice to expert) engaged with artwork (10, 42, 47). Bridging the gap between cognitive (neuro)science and art history (48, 49, 50), we present an eye tracking study with the aim to understand the interaction between image and text during the process of biblical art reception. The basic character of the study is an explorative one, which means that we use only a small number of participants and a few selected images from the illustrated Bible in order to study the interaction between text and image. More precisely, this study is aimed at the following questions. How are such (illustrated) Bible images perceived by today's recipients and do the accompanying texts attract the observer? And if so, at what time point does such attraction take place during the process of viewing? Further, is there any semantically driven interaction between image and text during the process of biblical art reception and how could such interaction be characterized? As a secondary objective, we demonstrate another successful collaboration between cognitive science & art history in an empirical study as proposed for future-oriented research towards ‘cognitive research in art history’ (50).

## Methods

### Participants

Ten participants were recruited for the study. All participants (5 males; age range: 22-28 years) were students at the University of Tübingen and naïve to the background and purpose of the experiment. Participants were recruited using notices in the buildings of the university and had normal or corrected-to-normal vision. Due to inadequate tracking quality (see below), two participants were excluded from the study. Thus, eight volunteers participated finally in the study.

### Apparatus

**Figure 1.**
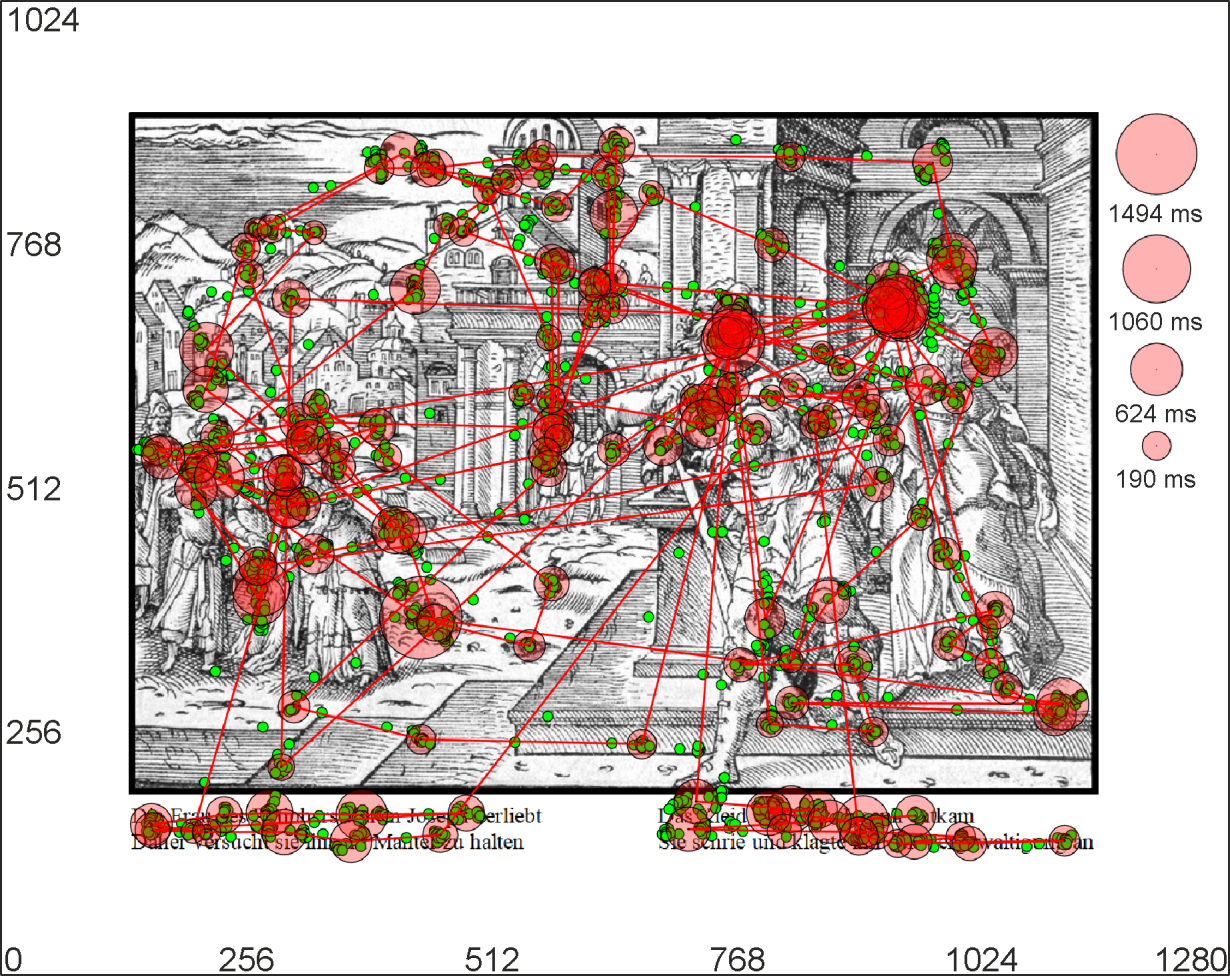
Example of a stimulus image (Image 4 | Genesis XXXIX) together with a superimposed, representative scanpath. The monitor’s margins together with dimensions are denoted as the outer black rectangle (1280 × 1024 pixels on the x/y axis, respectively). The white spacing of 140 pixel is apparent between margins and stimulus, enabling optimal eye tracking. An exemplary scanpath of a participant containing the spatio-temporal sequence of fixations (red circles with black edges) and saccades (red lines) is superimposed onto the stimulus. Raw data of the eye tracker (i.e. x/y coordinates) are shown as green dots. The calculated length of each fixation (i.e. fixation duration) is illustrated by the size of the red circle. Please refer to the legend on the right for the respective duration in milliseconds (the legend was not part of the stimulus).

Eye movements were recorded with a remote monocular eye tracker (Eyegaze Edge®, LC Technologies, Inc.) tracking the position of the participant’s left eye (in x/y coordinates related to the resolution of the monitor) with a temporal frequency of 60 Hz. The monitor used was a customary 19’’ monitor (screen size: 37.6 × 30.1 cm ≙ 1280 × 1024 pixel ≙ ±21.8° × ±16.7°; temporal frequency: 60 Hz) including white spacing of 140 pixels to the margins of the screen (cf. figure 1). This spacing was applied to enable optimal eye tracking within the appropriate area of the screen. The resulting size of the stimulus images was 1000 × 720 pixel (≙ ±16.4° × ±12.0°). Using a chin rest, the participant’s head was fixed, resulting in an eye-to-monitor distance of 50 cm. First, the eye tracking quality of each participant was tested. Because of inadequate tracking, two participants were excluded from further experimentation. The overall tracking quality of the eight participants included in the study was high (i.e. the average percentage of eye loss was 1.49 ± 0.93%; *x̅* ± *SD*). Immediately before the measurement, the eye tracker was calibrated using a standard 9-point calibration procedure. During data collection, eye tracking data (x/y coordinates in pixel) was stored as an ASCII data file after completion of each trial (i.e. viewing of the respective image). Fixations (and derived variables, see below) were analyzed based on the recorded raw data.

To extract fixations, a velocity-based algorithm was used: For each time step *t_0_*, a gliding window of 120 ms length centered at *t_0_* was considered. Let *v_min_* and *v_max_* denote minimal and maximal eye velocities obtained within the window. The instant *t_0_* is classiﬁed as belonging to a ﬁxation if *v_max_* - *v_min_* < 50 deg/s. This procedure is iterated through all time steps. Adjacent instants in time satisfying the condition are combined to ﬁxational events.

### Material

The four stimulus images were taken from a book from the genre of illustrated Bibles: *Neuwe Biblische Figuren, deß Alten und Neuwen Testaments* (New biblical figures of the Old and New Testament) published in 1569 in Frankfurt am Main (51). It comprises a title page, a dedication by the publisher Sigmund Feyerabend, a poem to the reader, 127 (3 fold-out) woodcuts, and a closing page. The woodcuts were created by Jost Amman (1539-1592) according to drawings by Johann Melchior Bocksberger (1530-1589) (52). The book’s format is oblong with the dimensions of 16 × 20.5 cm. All the artists and editors involved in this book were famous and respected during their times (53, 54). For this book, Bocksberger and Amman developed a style that was new and advanced for the viewers at the time. Characteristic features are the large, expressive figures in the foreground and a detailed and lively surrounding with additional scenes, figures and animals.

**Figure 2.**
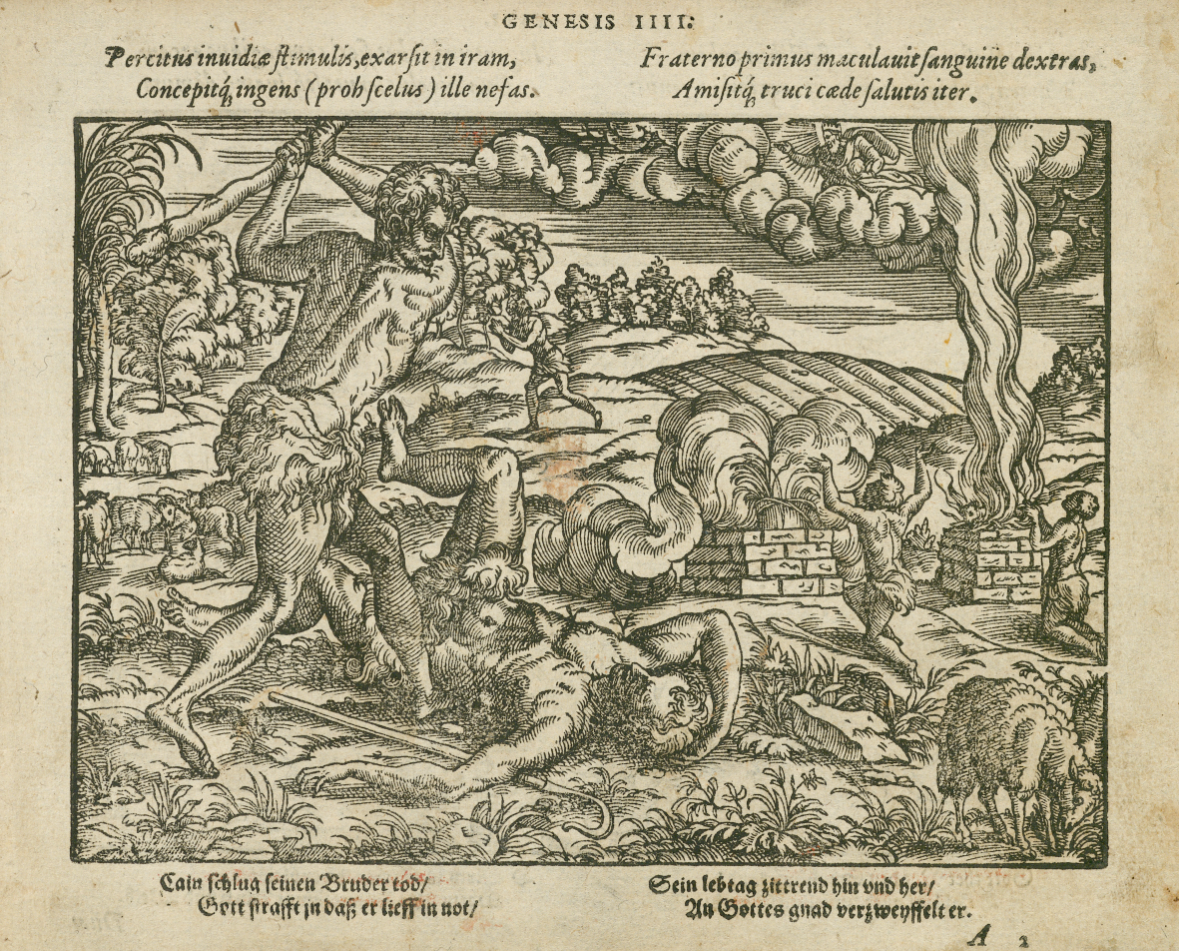
Original woodcut (Image 3 | Genesis IV (IIII)) from the illustrated Bible used for this study with the structure: heading - text (Latin) - image - text (old German). Please note that some images in this copy of the Bible show clear traces of use: some woodcuts are amateurishly coloured in ochre, red and green, although there is no knowledge about the time of this colouring jet. Such image disturbances were minimized using digital image processing (see text). Source of this woodcut: Johann Melchior Bocksberger und Jost Amman, Neuwe Biblische Figuren, deß Alten vnd Neuwen Testaments, Franckfurt am Mayn (Georg Rabe, Sigmund Feyerabend u. Weygand Hanen Erben) 1569, Folio 5r, Genesis IIII, Stuttgart, Württembergische Landesbibliothek (Inv. No. B graph. 156901). IMAGE © Württembergische Landesbibliothek.

The woodcuts show scenes from the Old and New Testaments and are combined with short texts whereby the image occupies a much larger space than the text (cf. figure 2). A headline indicates where the topic of the image can be found in the Bible (e.g. Genesis 2). Directly below the heading are four lines in Latin, which are repeated under the image in an old German version. The texts are short, rhymed paraphrases of the shown biblical narrative. This yields the following structure: Heading – text (Latin) – image – text (old German). The verses are divided, always showing two lines on the left and two lines on the right side.

**Figure 3.**
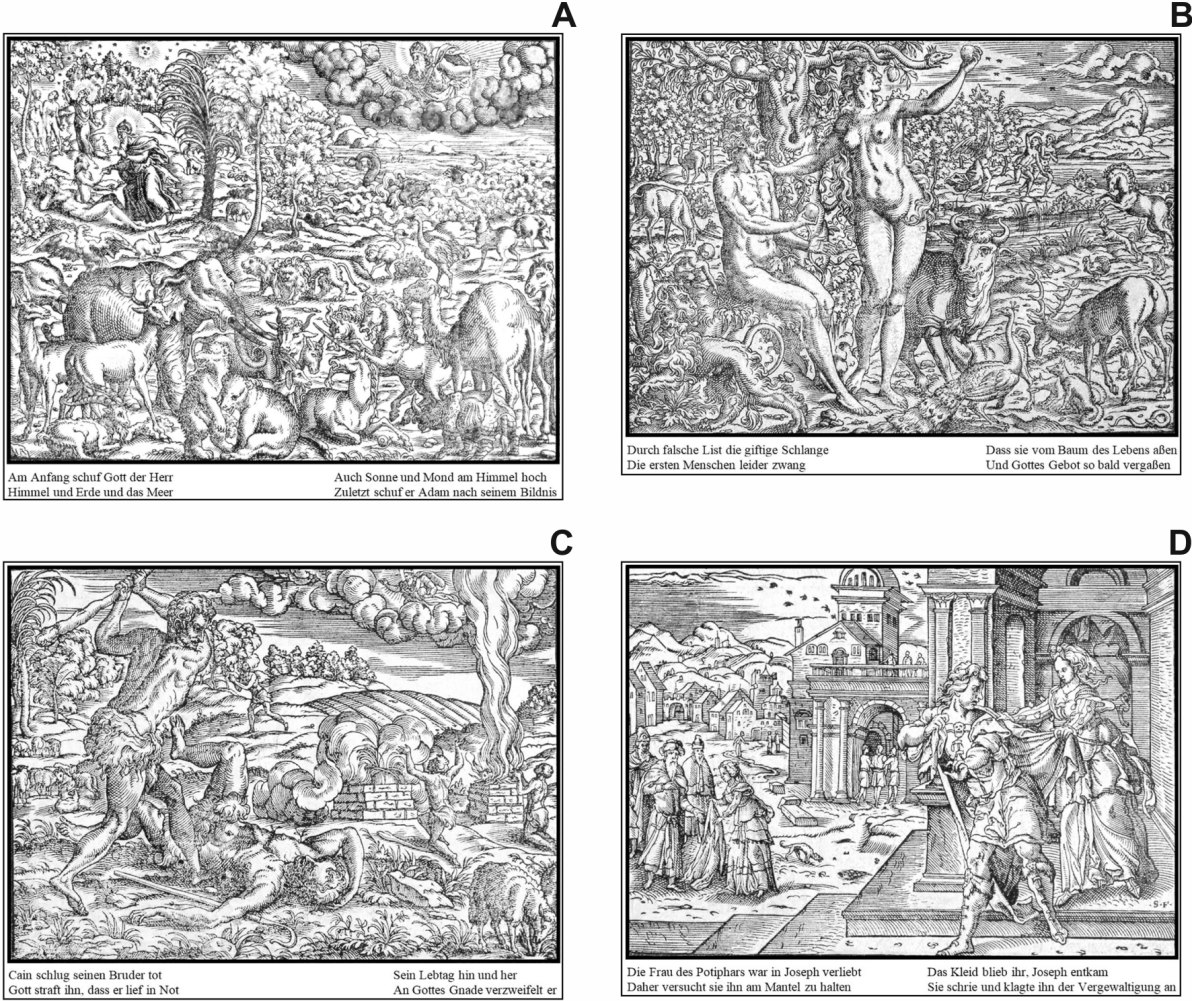
All stimulus images, which were used in the study (the practice image - Genesis VIII - is not shown): A) Image 1 | Genesis I (page 13), B) Image 2 | Genesis III (page 14), C) Image 3 | Genesis IV (IIII) (page 15), and D) Image 4 | Genesis XXXIX (page 27).

The four stimulus images used in the study were selected following these three criteria: i) the content of an image should be recognizable for a person who grew up in the European culture, ii) selected woodcuts should show a clear composition in which the protagonists can be identified by their size, gestures and activities, and iii) images should have a distinct and meaningful contextual relationship between text and image. In the chosen images (see figure 3), the text references the figures, the depicted scenes and the narrative:

- Image 1 | Genesis I (page 13) depicts the creation of Adam and Eve. Text in English: In the beginning, God the Lord created / Heaven and earth and the sea / Also sun and moon high in the sky / Lastly, He created Adam in his own image

- Image 2 | Genesis III (page 14) depicts Adam and Eve in the paradise. Text in English: Through false cunning the poisonous snake / Unfortunately, forced the first humans / That they ate from the tree of life / And so soon forgot God's commandment

- Image 3 | Genesis IV (IIII) (page 15) depicts Cain murdering his brother Abel. Text in English: Cain beat his brother to death / God punishes him so he ran into distress / His life-time back and forth / He despairs of God's grace

- Image 4 | Genesis XXXIX (page 27) depicts Joseph and Potiphar's wife. Text in English: Potiphar's wife was in love with Joseph / So, she tries to keep him by the coat / The dress stayed with her, Joseph escaped / She screamed and accused him of rape

To ensure the adequate perception of the images and readability of the texts, all images were processed using Adobe Photoshop CC (version: 2015.5.0). Initially, the images were cut out from the digitalized reference and converted to achromatic (grayscale) images. After some post-processing steps to increase and equalize their perceptual quality, the translated standard German texts were reinserted to generate the final stimulus images for the experiment (see figure 3). The Latin text of the original was omitted, because the participants could not read Latin and should not be confused, nor should their attention be distracted. The practice image (Genesis VIII) was processed without any text. This practice image was used to familiarize the participants with the stylistic features as well as the principal composition and contextual characteristics of the Bible images.

### Procedure

The whole experimentation was attained in a separate, dimly lit lab room where participants were not distracted by noises and suchlike. A personal computer (3.1 GHz) running MatLab 2018b (MathWorks Ltd.) was used for stimulus presentation, experimental control, and the recording of participants’ responses. The software controlling the experiment incorporated the Psychophysics Toolbox extensions (55). Initially, participants had to read a written task instruction. Here, they were instructed to look at each of the four images showing different narratives from the Bible for 60 seconds. They were expected to try to figure out the story behind the respective image in order subsequently report it to the experimenter (please note that the report of the stories narrative did not take place - this instruction was only meant to increase motivation and attention). Participants were never instructed in any way to use or attend to the text. After familiarization with the eye tracker and chin rest, the calibration procedure of the tracker was completed and the practice image was presented to the participant for 60 seconds. This image (Genesis VIII) was processed in the same way as the other stimulus images (see above) but presented without any text. The practice image was shown in order to familiarize the participants’ perception with the artistic style of the Bible images. After viewing the practice image, the four stimulus images (cf. figure 3) were presented in random sequence while eye movements were tracked. After each image, the tracker was calibrated again after a short break. The total duration of the experimentation was about 15 minutes.

### Design

The study followed a ‘within subject’ design, i.e., each participant had to process all four (plus practice image) images.

**Figure 4.**
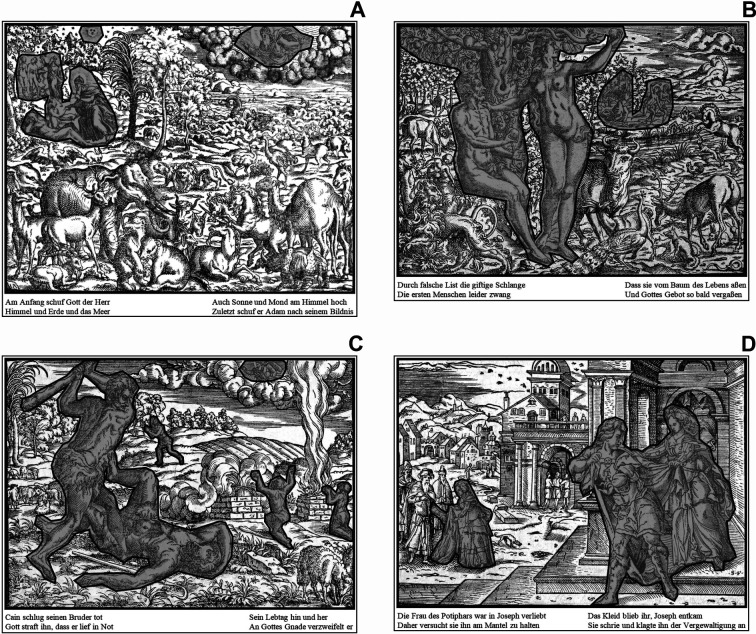
Pre-selected regions of interest (ROIs) for all four stimulus images (depicted in dark grey with a black outline).

Region of interest (ROI): In order to analyze the interaction between text and images, relevant ROIs (cf. figure 4) were a priori pre-defined for each image. Here, within the images, regions were chosen of which the contents were directly linked to the text (i.e. reflecting the narration and the most relevant figures, objects, and their interactions).

Measures: Based on the extracted fixations and their durations, a scan-path (i.e. the spatio-temporal sequence of fixations and saccades) was calculated for each participant and image and superimposed statically (see figure 1) or dynamically to the stimuli. The dynamic method resulted in an animated temporal sequence of the spatial positions of all fixations and their durations. These animations were recorded as movie files for the ROI analysis. To quantify the general use of text, three variables were calculated. The *frequency of looking at text* measures how often the text was scanned (read) during the 60 s of stimulus observation (i.e. the number of text-scanning periods). The *initial number of fixation* counts all fixations before the first text-scanning period. The *number of fixations on text* quantifies all fixations that were part of text-scanning periods (i.e. if a text was scanned several times, all according fixations were added up).

To assess the relevance of ROIs together with the interaction of text and image, three further variables were calculated. The *overall proportion of fixation in ROI* measures the proportion of all fixations within the 60-second observation period that were directed to ROIs. The first ten consecutive fixations immediately after the first text-scanning period were analyzed and the *proportion of fixations in ROI after text* counts how many of these ten fixations were directed to ROIs. Note that if less than ten fixations occurred between two subsequent text-scanning periods, the initial fixations directly after the second text-scanning period were added to make up the ten fixations. For the variable *proportion of longest fixation duration in ROI*, the ten fixations with the longest duration (fixations on text were ignored) were calculated. Then, the proportion of how many of these longest fixations were fixations on ROIs was calculated.

**Figure 5.**
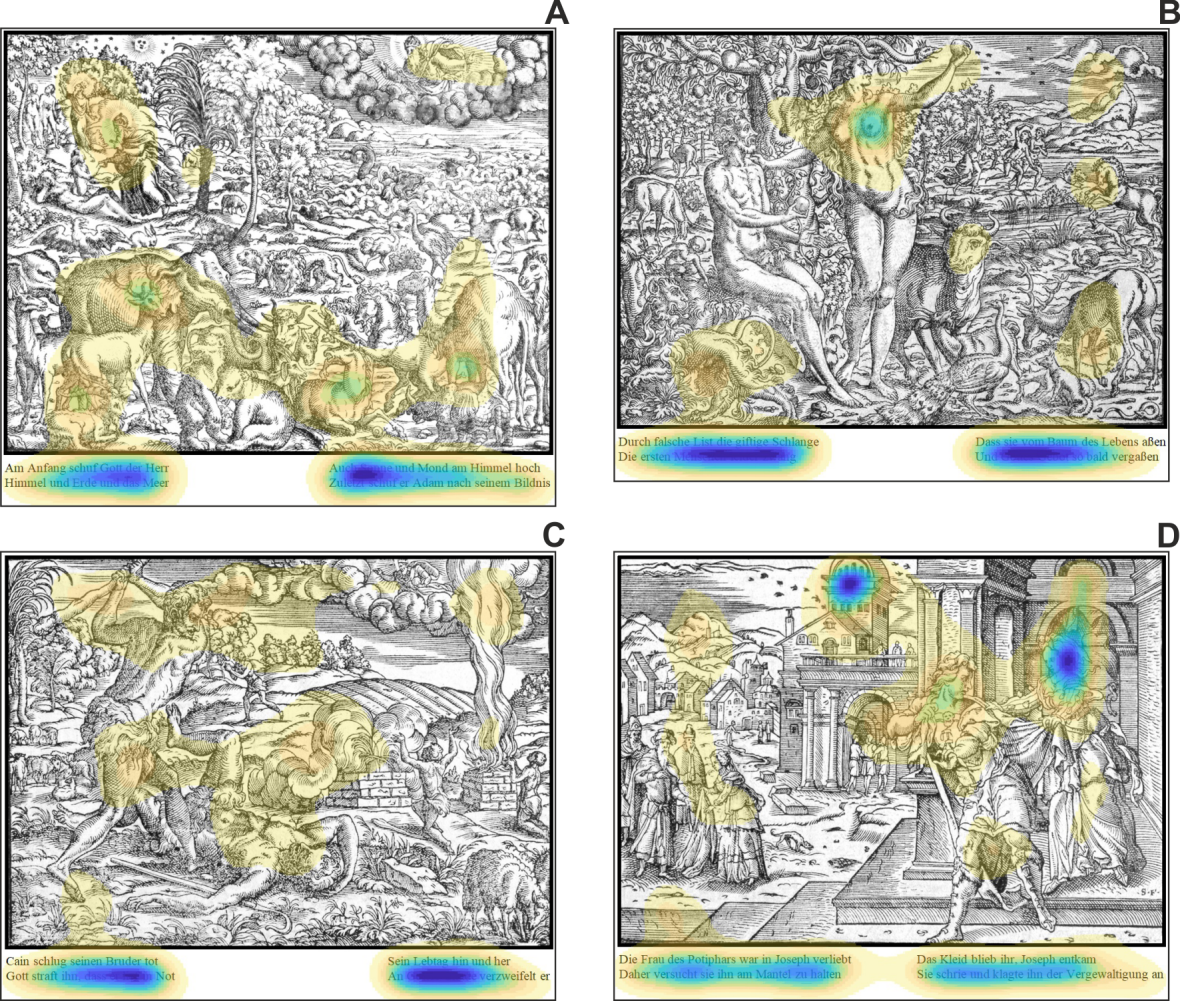
The four stimulus images, which were used in the study are shown with the superimposed modelled saliences. Saliencies were calculated using the DeepGaze II algorithm. The probability distribution of fixations over the image are visualized as a color map using 15 color levels (from dark blue over yellow to transparency). The colors separate the image into 15 areas of decreasing probability density (dark blue depicts the highest density) such that each area has the same total probability mass.

Saliency modelling: In order to calculate and visualize saliencies in our images, we used the DeepGaze II saliency model (56). DeepGaze II is a state-of-the-art saliency model for predicting fixations in images based on saliencies (bottom-up processing). The model uses deep neural networks and makes use of convolutional ﬁlters that have been learned on other tasks, most notably object recognition (57, 58). The saliency prediction of such a model suggests that the high-level image features encoded by deep networks (e.g. sensitivity to faces, objects and text) are extremely useful to predict human ﬁxation locations (e.g. (59)). The DeepGaze II algorithm does not model top-down inﬂuences such as task or semantic properties, but rather predicts to what extent ﬁxations in free viewing are driven by low- and high-level features of the image. The outcome of the used DeepGaze II algorithm (56) is a probability distribution of potential fixations over the image. To visualize the densities, the continuum of distribution values was transformed in a color map with 15 color levels and overlaid with the stimulus image (see figure 5). The colors separate the image into 15 areas of decreasing probability density such that each area has the same total probability mass (i.e. the density predicts each area to receive the same number of ﬁxations).

Statistics: The test for significance of the presented variables was accomplished using the non-parametric Friedman Test, because the total sample size was not large enough to demonstrate a normal distribution. Post-hoc effects were calculated using Dunn’s pairwise post hoc tests with Bonferroni correction. As measure for the size of an effect, Kendall’s W (Coefficient/Degree of concordance) was calculated. Kendall’s W is a test which looks at agreement between participants and gives a value which ranges between 0 and 1. Kendall uses the Cohen’s interpretation guidelines of 0.1 (small effect), 0.3 (moderate effect), and above 0.5 as a strong effect. All statistics were calculated using SPSS (version 25; IBM Corporation, New York, USA).

## Results

Using state-of-the art saliency modeling, we calculated and visualized the distribution of predicted fixations for the four biblical images (see figure 5). Again, the modeled saliencies purely based on bottom-up processing without any contribution concerning top-down mechanisms (see above).

**Figure 6.**
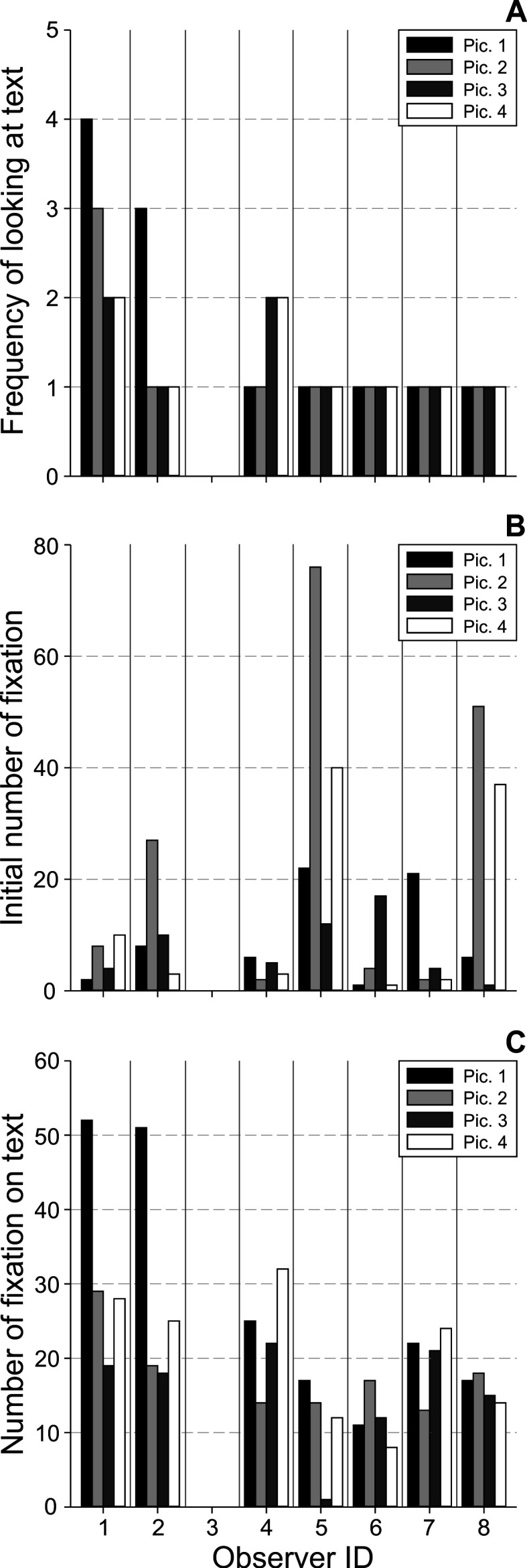
Measures related to the overall use of text: A) Frequency of looking at text, B) Initial number of fixation, and C) Number of fixation on text analyzed for each observer (abscissa) and image 1-4.

**Figure 7.**
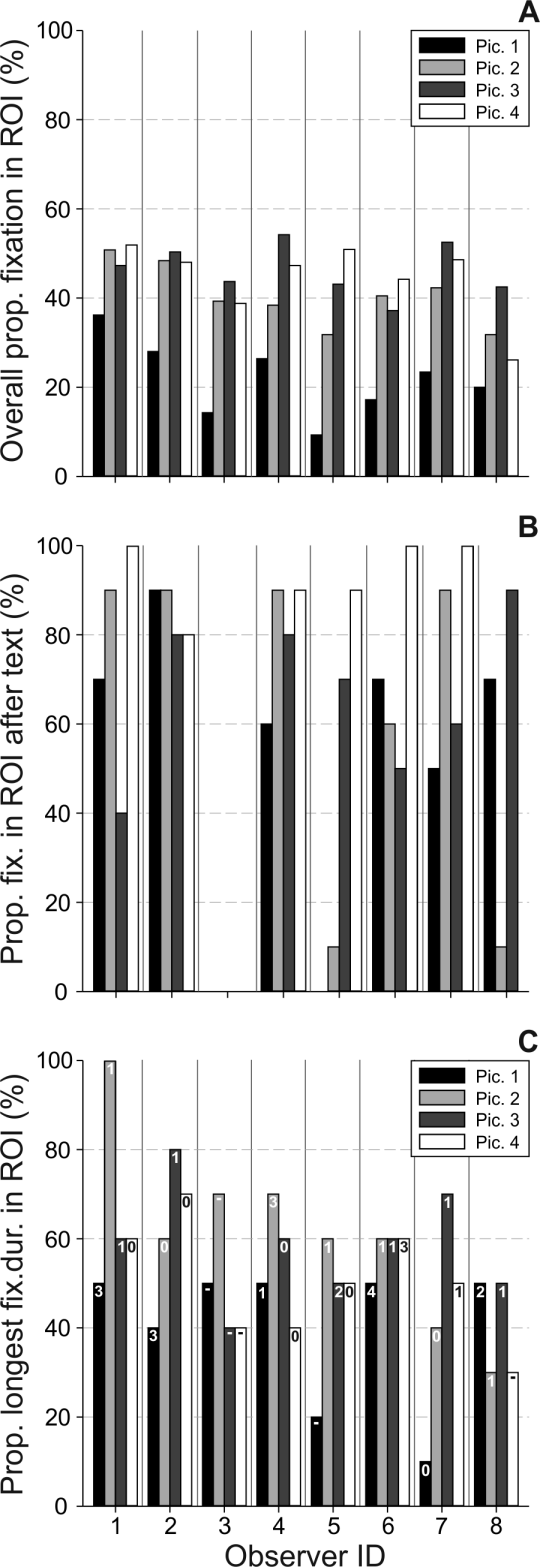
Measures related to the relevance of ROIs & text interaction: A) Overall proportion of fixation in ROI, B) Proportion of fixations in ROI after text, and C) pro-portion of longest fixation duration in ROI (numbers indicate the amount of longest fixations, which occurred directly after text-scanning) analyzed for each observer (abscissa) and image 1-4.

In order to quantify and characterize the interaction between image and text during the process of art reception, the analysis of eye movement data comprises two steps. In the first step of analysis, the overall use of text was investigated to identify the importance of the texts for each participant and stimulus image (figure 6). In the following, the overall attention to ROIs as well as the interaction between text and ROIs immediately after the text-scanning and the meaning of fixation duration and ROIs was investigated (figure 7).

### Overall use of text

The average *frequency of looking at text* over all participants and images was 1.39 ± 0.78 (*x̅* ± *SD*), showing that at least one text-scanning period was performed per participant and image (figure 6A). The most frequent use of texts was shown by participant 1 (2.75 ± 0.95) and absolutely no fixating of texts was found for participant 3. The majority of observers performed just one text-scan for each of the four stimulus images. There was no significant effect of observer (*χ*^2^(6) = 11.63, *p* = 0.072) or of image (*χ*^2^(3) = 1.44, *p* = 0.7) on *frequency of looking at text*. To analyze the temporal importance of texts during the process of reception, the number of fixations, prior to the first text-scanning period (i.e. *initial number of fixation*), was quantified (figure 6B). Here, participants spent on average 13.75 ± 17.9 fixations (*x̅* ± *SD*) on initial parts of the image before the text was deemed important. Interestingly, while five participants (ID 1, 2, 4, 6, and 7) showed overall low numbers of initial fixations (below 7), participants 5 and 8 showed a remarkably high number of fixations for the images 2 and 4 (above 38).

**Figure 8.**
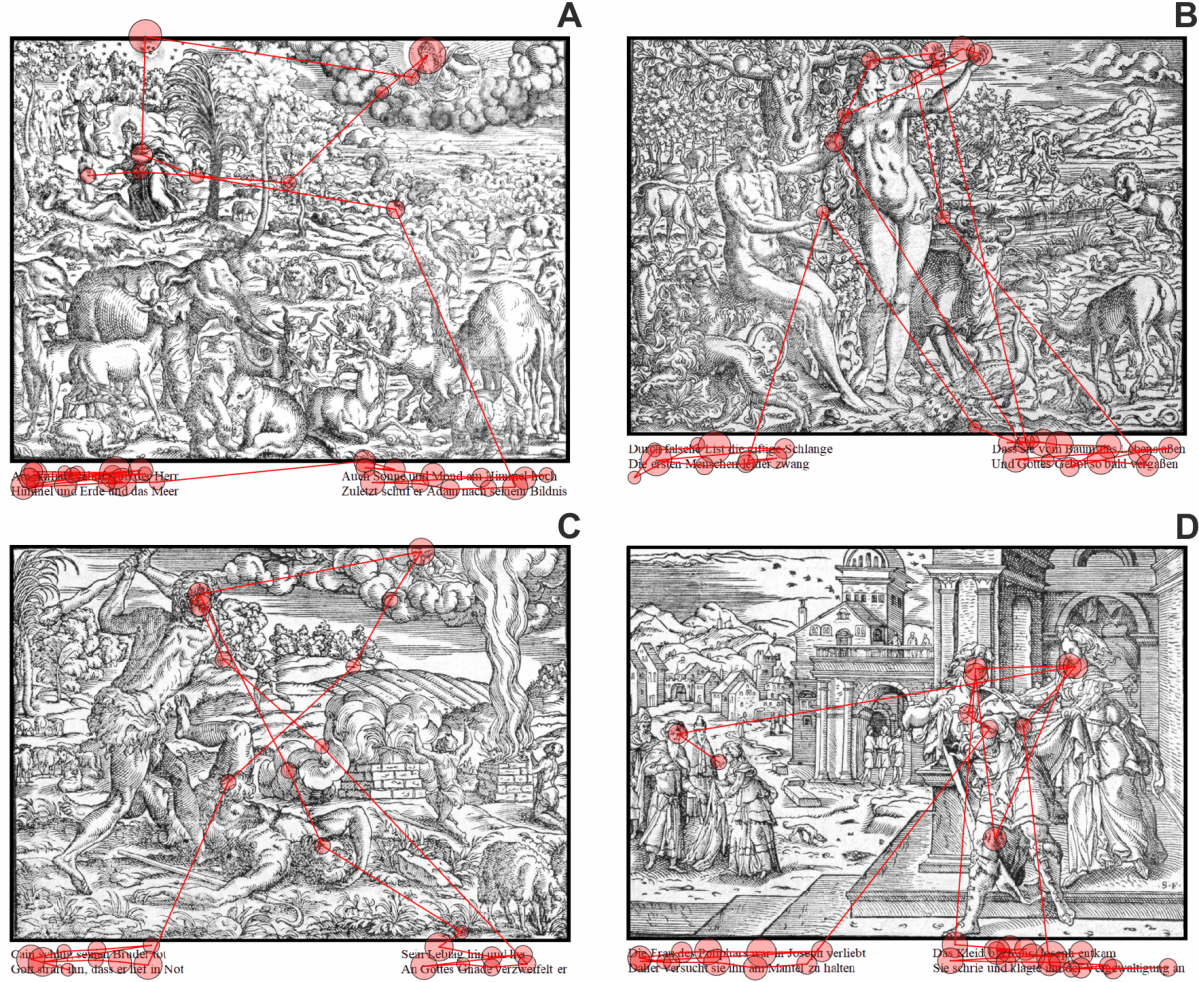
Partial scanpaths of an exemplary observer (ID 1) shown for all stimulus images: A) Image 1 | Genesis I (page 13), B) Image 2 | Genesis III (page 14), C) Image 3 | Genesis IV (IIII) (page 15), and D) Image 4 | Genesis XXXIX (page 27). The first ten fixations following the initial text-scanning period are plotted together with the complete scanning of the text. The scanpaths contain the spatio-temporal sequence of fixations (red circles with black edges; size of the red circles illustrates fixation duration) and saccades (red lines).

Participant 3 was not analyzed for this measure, since no text-scanning period was found. There was neither a significant effect of observer (*χ*^2^(6) = 9.7, *p* = 0.14) nor of image (*χ*^2^(3) = 1.1, *p* = 0.78) on *initial number of fixation*. The last measure to characterize the usage of text quantified the number of fixations spent while looking at texts (i.e. *number of fixations on text*). This measure is important for interpreting the attention to and perception of texts and thus, the relevance of the text-scanning periods. The average number of fixations on text was found to be 20.35 ± 11.02 (*x̅* ± *SD*), showing that text-scanning was applied to read the texts and understand their meaning (figure 6C). Except for the single text fixation of participant 5 on image 3, all other text-scanning periods included a sufficient amount of fixations (and their dispersion; cf. figure 8 (parts of scanpaths of ID 1)) necessary for text reading. There was a significant overall effect of observer on *number of fixations on text* (*χ*^2^(6) = 14.68, *p* = 0.023, Kendall’s *W* = 0.611), but no meaningful post hoc effects could be observed. There was no significance of image on *number of fixations on text* (*χ*^2^(3) = 5.23 *p* = 0.16).

### Relevance of ROIs & text interaction

In order to analyze the overall salience and importance of the ROIs, the proportion of all fixations directed at the ROIs of the images (irrespective of text-scanning periods) was quantified (i.e. *overall proportion of fixation in ROI*; see figure 7A). There was a significant effect of observer on *overall proportion of fixation in ROI* (*χ*^2^(7) = 17.03 *p* = 0.017, Kendall’s *W* = 0.608). Also, there was a statistically significant difference in *overall proportion of fixation in ROI* depending on which type of image was presented (*χ*^2^(3) = 15.75, *p* = 0.001, Kendall’s *W* = 0.656). Individual averaged values (*x̅* ± *SD*) for image 1 to 4 are: 21.85 ± 8.51, 40.41 ± 6.85, 46.36 ± 5.76, 44.48 ± 8.48%. Statistically, there was an increase in the percentage of fixations in ROIs from image 1 to 3 and 1 to 4 (note that this is not a sequence effect of image presentation, since images were presented in random order). These individual values are also always higher than the proportion of the pure area of the ROIs per image (1: 10.2, 2: 35.05, 3: 28.05, and 4: 26.95%; cf. figure 4) showing an enhanced functional meaning of the ROIs as opposed to just random observation. This enhanced meaning was further highlighted when solely the ten fixations performed immediately after the first text-scanning period were considered (i.e. *proportion of fixations in ROI after text*; see figure 7B).

Here, on average, 69.63 ± 27.9 (*x̅* ± *SD*) percent of fixations were identified within the areas of ROIs, showing an increase in the attraction of elements within the ROIs immediately after reading the text of an image.

Since the majority of observers scanned the text only once (cf. *frequency of looking at text*; figure 6A), the variable *proportion of fixations in ROI after text* includes just the initial text-scanning period(s). There was no significant effect neither of observer (*χ*^2^(6) = 3.5, *p* = 0.75) nor of image (*χ*^2^(3) = 6.74, *p* = 0.08) on *proportion of fixations in ROI after text*. To visualize and highlight the text-image interaction further, the partial scanpaths of an exemplary observer (i.e. ID 1) are shown in figure 8. Here, the first ten fixations following the initial text-scanning periods were plotted for all four stimulus images together with the complete scanning of the text. Interestingly, the majority of these fixations was directed to the ROIs (i.e. 70, 90, 40, 100% for image 1 to 4, respectively).

As a further measure supporting the increased importance of the ROI-content, the *proportion of longest fixation duration in ROI* was calculated (figure 7C). On average, 52.5 ± 17.41 (*x̅* ± *SD*) percent of the longest fixations were identified as being within the ROIs. There was neither a significant effect of observer (*χ*^2^(7) = 9.01, *p* = 0.25) nor of image (*χ*^2^(3) = 6.97, *p* = 0.073) on this measure. Note that only a minority of the longest fixations occurred directly after text-scanning, as indicated by the number of the longest fixations as part of the measure *proportion of fixations in ROI after text*.

## Discussion

In the current study, we let naïve observers to look at images showing biblical scenes with accompanying texts and analyzed their gaze behavior. The task of the observers was simply to make out the story behind the biblical scenes, i.e. the narration. We conducted this exploratory study to investigate and understand, how (and if) the text is used in such a process of understanding narration.

### Stimulus saliencies

The analysis of saliencies clearly shows that the texts are the most attractive elements in all images, i.e. the dark blue color indicates their highest probability density. Such finding is to be expected, since the DeepGaze II saliency model has also learned to incorporate text as high-level (feature) object (59). With a much lower amount of probability density, regions within the images were quantified. Some of these regions overlap with the ROIs, which we have chosen because of their importance in reflecting the narratives. However, identified saliencies also highlight regions that have no reference to the narrative supported by the text. For instance, the lower area of image 1 (Genesis I) was rated as salient, but the highlighted objects (animals; see figure 5A) were not part of the semantical content of the text.

### Overall attraction of texts

The texts provided with the biblical images undoubtedly attracted the observer’s attention and led them to read each text at least one time (i.e. average *frequency of looking at text*: 1.39) and almost immediately after stimulus onset (i.e. average *initial number of fixation*: 13.75). Two observers performed more than just one reading phase (ID 1 and 4). Such repeated interest might support the finding of the overall high attraction of texts. Once a text was viewed, observers actually read it, as demonstrated by the overall high *number of fixations on text* (on average: 20.35) as well as by the lengths of these fixations (i.e. average fixation duration: 328 ± 85 ms; *x̅* ± *SD*) showing the meaningful value of reading behavior (60). The majority of observers performed just one text-scanning period for a given stimulus image and showed only a small number of initial fixations. Except for participant 3 and for the images 2 and 4 also participants 5 and 8, all observers showed a very similar text-scanning behavior: After some initial fixations within the area of the image (between 2 and 25), participants gazed at the text, read it, took in its narration, and attended to the image again utilizing the information provided by the text (see below, next chapter). The initial phase of image scanning is most likely needed to extract the coarse ‘structure’ of the scene, i.e. knowledge about the scene gist of an image (41, 61, 62). Besides creating such a scene gist, the initial scanning could additionally serve to preview ‘salient’ objects and possible targets, subvocalize them, and thus pre-generate the linguistic labels that may appear later in the text (20, 60). After creating such an over- or preview participants subsequently read the text and extracted the semantic as well as contextual information from this ‘instruction’ (13, 14, 22). It can be concluded that the recipients have really read the text (and thus understood its narration) by looking at the number and the duration of the observed text-scanning fixations together with the observed meaningful reading pattern (i.e. participants started text-scanning at the top left and always either scanned the complete text at once or first scanned the left two lines and then the right two lines of the text; cf. figure 1). Similar to our interpretation, Leder et al. (36) suggest two processing stages during the aesthetic engagement and judgement with artworks: an early automated and a later deliberate one. The early processes include perception of form, recognition of objects, and the extraction of content. During the deliberate processing phase, observers may engage in an interpretative process by explicitly employing declarative knowledge to attribute iconological contents to the artwork (36). Here, iconographic content is the classification of the preiconographic contents according to cultural interpretation conventions (43).

Interestingly, some participants deviate from the general pattern of text use insofar that they either never looked at the texts (ID 3) or showed very prolonged durations regarding the initial phase of image scanning (ID 5 and 8, images 2 and 4; i.e. between 37 and 76 initial fixations; cf. figure 6B). Since we did not interview the participants after experimentation, we do not know if observer 3 came up with the same narrations of all biblical scenes compared to the others. Participants 5 and 8 (despite partly long initial scanning) nevertheless showed a very similar use of texts together with a conclusive reading pattern as seen by the *number of fixations on text* (cf. figure 6C) and the scanpaths.

In conclusion, our data show the clear attraction of texts in almost all stimulus images and that the texts attract the gaze in an early state of inspecting the illustrated Bibles. Furthermore, if the texts were noticed, they were carefully read and understood.

### Interaction between image and text

The most interesting aim of this study was to understand the possible (semantically driven) interaction between the read text and the following inspection of the biblical image. In a first step, we quantified the overall appeal of the relevant elements in the images by measuring the *overall proportion of fixation in ROI* irrespective of text-scanning. The analysis of this measure revealed i) an overall moderate attraction rate of the ROIs (i.e. on average 38.25% of all fixations landed in ROIs) and ii) an increase in the percentage of fixations in ROIs from image 1 to 3 was found for all observers. Since the proportion of the pure area of the ROIs per image could not explain such an increase, the feature-driven salience (bottom-up processing) or the observer’s experience and pre-knowledge (top-down processing) may be responsible for the varying overall attraction of the ROIs.

Together with the *overall proportion of fixation in ROI,* the *proportion of longest fixation duration in ROI* was analyzed. Similar to the spatial relevance of fixation distribution, their temporal characteristics also support an overall appeal of the ROIs. Irrespective of text-scanning, the *proportion of longest fixation duration in ROI* was remarkably high with an average of about 52 percent. Taking both measures together shows the overall meaningful ‘salience’ or attraction of the ROI-contents largely irrespective of the contextual information carried by the texts. The text-independent attractiveness of ROIs is further confirmed by observer 3 - although never gazing at the texts, this participant has similar values for the *overall proportion of fixation in ROI* and the *proportion of longest fixation duration in ROI* compared to the other observers.

The most interesting measure concerning a semantically driven text-image interaction was the proportion of fixations in ROI immediately after having read the text. Here, a significant increase in comparison to the *overall proportion of fixation in ROI* was found for each image. The values (*x̅* ± *SD*) for the *proportion of fixations in ROI after text* for image 1 to 4 are: 58.57 ± 28.54, 62.86 ± 37.73, 67.14 ± 17.99, 93.33 ± 8.16% (i.e. on average about 70 percent). Such high proportions reveal the increased attraction of the elements in ROIs as soon as observers have the narration and contextual information provided by the text in mind. However, this increased spatial attraction of elements in ROIs is not accompanied by a prolonged temporal fixation pattern (i.e. only a very few of the longest fixations occurred directly after text-scanning; cf. figure 7C, numbers in the plot).

A more detailed investigation of the spatio-temporal pattern of the ten fixations immediately following the text-scanning impressively shows the strong interaction between text and image. A representative example of this spatio-temporal pattern is provided in figure 8. Here, fixation targets comprised not only the faces of persons in ROIs, as their bodies and pose also attracted the observers while they had previously read textual information in mind (cf. figure 8). Faces are obviously important because they carry information about the emotion of the person and their social interaction with others (63, 64). Fixations on bodies and their pose are necessary to be able to infer about the agent’s state of action as well as the physical action (and embodied situation) in which the person is involved (32, 65). Figure 8 also very nicely shows that observers are able to comprehend the narration of the story, i.e. their fixational pattern is not only driven by the elements mentioned in the text, since also the interactions and intentions of these elements are represented in the spatio-temporal distribution of overt attention (cf. also the complete scanpath in figure 1). In conclusion, the partial scanpaths of our participants (except number 3, which showed no text scanning) show very convincingly that i) the text was scanned appropriately and ii) that objects (and probably also their relations) mentioned in the text were attended immediately after reading the text. These scanpath data indicate with high probability that our participants comprehended the text and used the processed content to fixate relevant elements of the image. With this first study on text-image interactions in biblical art, we were able to show that today’s viewers use the accompanying texts to better connect with the image. The degree of transferability of our findings to 16th century viewers cannot be determined. However, since it is reasonable to assume that visual functions (i.e. oculometric control, overt|covert attention, salience vs. task-driven gaze pattern, etc.) between the people of that time have not changed essentially in comparison to today, it can be assumed that Bible readers in the 16th century also used the provided texts when viewing illustrated Bibles. Empirical research can therefore support and stimulate historical observer research and shed light on ambiguous passages in the sources.

The results of our exploratory study convincingly point to promising research on gaze movements for the understanding of text-image interactions in art. Future studies should consider the following improvements: i) a higher number of participants together with different groups (in terms of age and expertise), ii) a more controlled stimulus material in the sense that ROIs are rather homogenous and numerous. With such improvements, more intensified analyses regarding the text-image interactions, e.g. characteristics of saccades within vs. between ROIs, number and distribution of transitions between ROIs, and the influence of the level of expertise (declarative knowledge) could be applied.

### Ethics and Conflict of Interest

The author(s) declare(s) that the contents of the article are in agreement with the ethics described in http://biblio.unibe.ch/portale/elibrary/BOP/jemr/ethics.html and that there is no conflict of interest regarding the publication of this paper.

### Acknowledgements

We wish to thank Tanja Koch from the University of Tübingen for her help in running the experiments.
